# Trends in allergen-reactive CRTH2+ T cells and TARC associated with successful outcomes in a phase 2 cashew oral immunotherapy study

**DOI:** 10.3389/fimmu.2025.1655975

**Published:** 2025-10-22

**Authors:** Sayantani B. Sindher, Andrea Fernandes, Monali Manohar, Shu Cao, Sheena Gupta, Ella Parsons, Dinara Bogetic, Divya Kumar, Jessica Rogers, Julia Thompson, Diane Dunham, Evan Do, Sofia Maysel-Auslender, Taryn Audrey Liu, Kristine Martinez, Brent Anderson, Abhinav Kaushik, Manisha Desai, Holden Maecker, Susan Perry, Lisa M. Wheatley, Kari C. Nadeau, R. Sharon Chinthrajah

**Affiliations:** ^1^ Sean N. Parker Center for Allergy and Asthma Research, Department of Pathology, Stanford University School of Medicine, Stanford, CA, United States; ^2^ Human Immune Monitoring Center, Institute for Immunity, Transplantation, Infection, Stanford University School of Medicine, Stanford, CA, United States; ^3^ Department of Environmental Health, Department of Microbiology and Immunology, Stanford, CA, United States; ^4^ Department of Environmental Health, Harvard T.H. Chan School of Public Health, Boston, MA, United States; ^5^ Quantitative Sciences Unit, Stanford University School of Medicine, Stanford, CA, United States; ^6^ Division of Allergy, Immunology and Transplantation, National Institute of Allergy and Infectious Diseases, National Institutes of Health, Bethesda, MD, United States

**Keywords:** food allergy, oral immunotherapy, allergen-specific T cells, cashew allergy, tolerance

## Abstract

**Background:**

We designed an oral immunotherapy (OIT) clinical trial for cashew allergy to further our understanding of immunological responses with treatment, including changes in allergen-specific T cells. This information can further assist with the design of efficacious and safe treatments.

**Methods:**

Participants were built up to and maintained on 1 g of cashew flour protein. Double-blind, placebo-controlled food challenges (DBPCFCs) were conducted before and after dosing completion (week 52) and 6 weeks after dosing discontinuation (week 58). Desensitization (DS) and sustained unresponsiveness (SU) were defined as tolerating DBPCFC to a cumulative dose of 2043 mg of the allergen at weeks 52 and 58, respectively. ClinicalTrials.gov, number NCT03504774.

**Results:**

We enrolled 40 cashew allergic participants. In the Intent-to-treat (ITT) population, both the DS and SU rate to cashew was 65% (26/40). Among cashew-reactive cells, CRTH2^+^ CD4^+^ T cells decreased at week 52 and week 58 compared to baseline. Additionally, we also saw reduced baseline expression of cytokines TARC, EGF and IP10 among participants that achieved SU at 4043mg compared to those who achieved SU at 2043mg.

**Conclusion:**

Cashew OIT have efficacy and safety outcomes similar to other published OIT studies. Reductions in pathogenic allergen-specific T cell populations may contribute to the immune mechanisms underlying tolerance achieved towards cashew post-treatment.

**Clinical trial registration:**

ClinicalTrials.gov, identifier NCT03504774.

## Introduction

Food allergy is a major global health concern. Peanut oral immunotherapy (OIT) has a well-characterized safety and efficacy profile but there remains a need to extend this knowledge on OIT to other allergens. Prior studies have shown that severe anaphylaxis reactions are strongly associated with cashew sensitization (1) and cashew allergies contribute to substantial societal and economic burdens associated with food allergies. Thus, advancing oral immunotherapy for cashew allergies will enhance patient outcomes. Furthermore, OIT is not universally successful for any food allergen and better insight into the mechanisms responsible for the success or failure of OIT for any food allergen could potentially enhance the prospect for better OIT.

**Figure 1:**
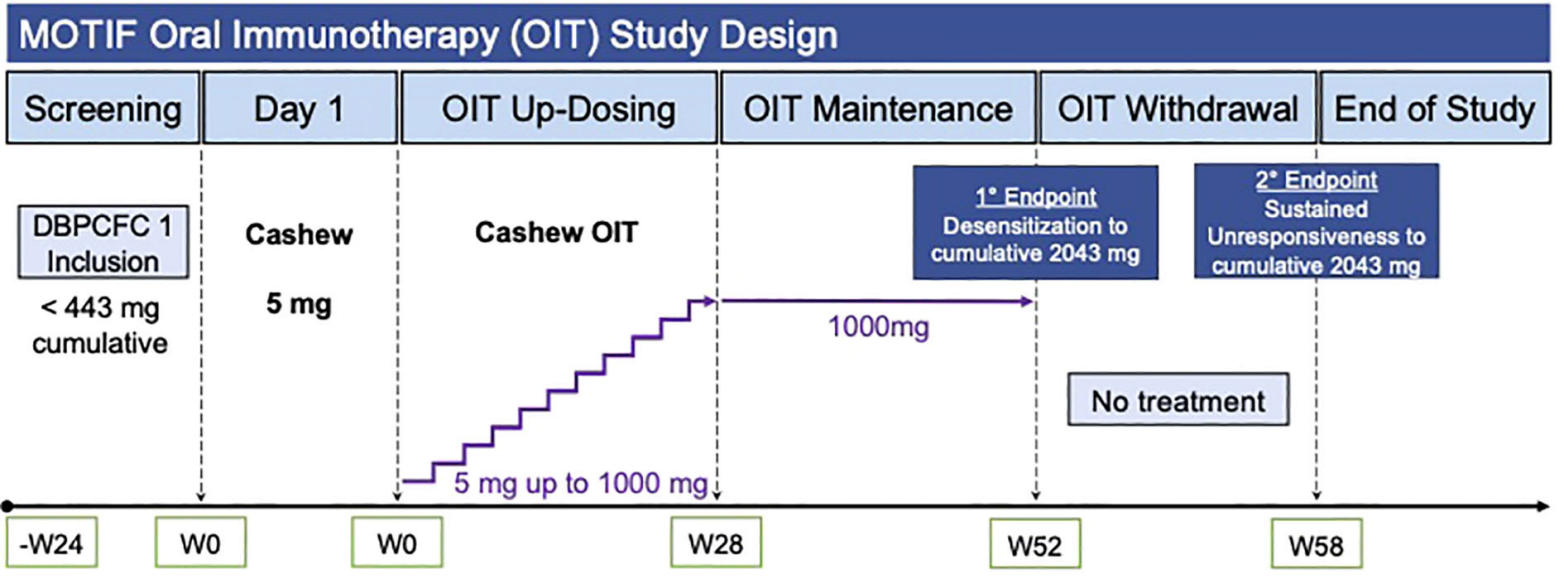
MOTIF study design and workflow. Legend: OIT, Oral Immunotherapy; DBPCFC, Double-Blind, Placebo-Controlled Food Challenge.

We hypothesized that OIT for cashew allergies would elicit immune tolerance by reducing allergen-specific CD4+ T cells, particularly pathogenic CD4+CRTH2+ Th2A cells. Toward this end, we conducted an open-label, Phase 2 clinical trial for participants who have confirmed allergies to cashew. Participants underwent 52 weeks of OIT followed by 6 weeks of avoidance of the offending allergen. Oral food challenges were performed at baseline, post-OIT (Week 52) and post-avoidance (Week 58) (**Figure 1**). Participants were assessed by performing oral food challenges for their ability to achieve allergen desensitization (DS) and sustained unresponsiveness (SU). Given the ethical considerations, a placebo group was not included, as withholding a potentially effective treatment was deemed inappropriate by the Institutional Review Board (IRB) due to the established efficacy of OIT for similar allergens. We additionally investigated allergen-specific phenotypes through immunophenotyping of humoral and T-cell responses before, during and after therapy to look for correlates of success. We performed immunophenotyping to explore allergen-reactive T-cell populations to identify potential biomarkers indicative of successful therapy and long-term immune tolerance. We also measured cytokine profiles to explore their role in promoting immune tolerance. By examining these immune parameters, we aimed to gain insights into the biological processes driving the clinical outcomes observed in our participants.

As expected, OIT induced a decrease in cashew-specific IgE and an increase in cashew-specific IgG4 over the course of treatment. This was accompanied by a decrease in the skin prick test wheal diameter, indicating reduced allergic reactivity. Additionally, these changes were associated with a reduction in allergen-reactive CD4+ T cells and lower levels of pro-inflammatory cytokines at baseline among participants who achieved SU to a higher dose of the allergen (4043 mg) compared to those who only achieved SU to a lower dose (2043 mg). These results suggest that OIT induces immunological changes, including modulation of IgG4/IgE ratio and suppression of allergen-specific T cell responses in cashew allergic participants, that underlie clinical DS and potential for achieving long-term SU in food-allergic individuals.

## Methods

### Study design

We conducted a prospective phase 2, single- allergen OIT study (MOTIF, NCT03504774 ) of cashew or shrimp at the Sean N. Parker Center for Allergy and Asthma Research at Stanford University (Stanford, CA, USA), with Stanford IRB approval under IND 18892. The clinical research protocol (Appendix 1) was reviewed by the Division of Allergy, Immunology, and Transplantation (DAIT) and the National Institute of Allergy and Infectious Diseases (NIAID) Allergy and Asthma Data Safety Management Board, the Stanford Institutional Review Board, and the US Food and Drug Administration (FDA). Although the trial included both cashew and shrimp OIT, this manuscript focuses on the findings for cashew allergy. The original protocol was amended to be more inclusive and to clarify risk and safety precautions. The study was done in conformity with the current revision of the Declaration of Helsinki and with the International Conference for Harmonization Good Clinical Practice (GCP) regulations and guidelines.

### Participants

**Figure 2:**
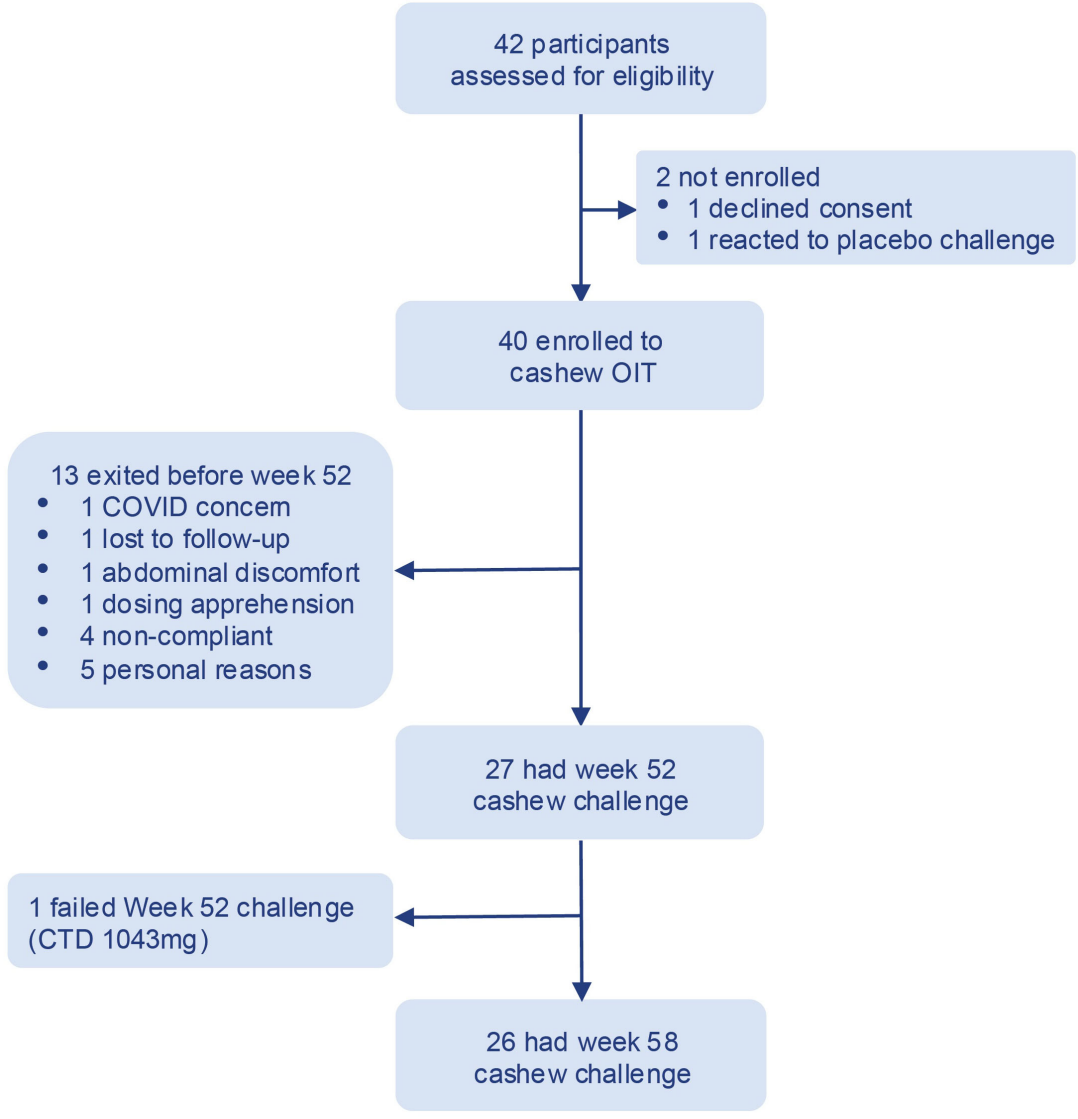
CONSORT Diagram.

Participants aged 7 to 55 years, meeting the inclusion and exclusion criteria for cashew allergy based on a screening protocol within the past 40 weeks, were recruited for the study. A total of 42 individuals provided consent and underwent eligibility assessment, leading to 40 cashew participants eventually enrolling in the study. Among those not enrolled, 1 withdrew consent, and 1 screen failed (reacting to the placebo challenge) (**Figure 2**).

Inclusion criteria comprised a clinical history of cashew allergy, serum IgE to cashew ≥ 0.35 kUA/L, and/or a skin prick test (SPT) to cashew ≥3 mm compared to diluent control, and dose-limiting symptoms at or before the 300 mg dosing level of food allergen (FA) protein on Screening Double-Blind Placebo-Controlled Food Challenges (DBPCFC). Exclusion criteria included severe or uncontrolled asthma, a history of uncontrolled cardiovascular disease, eosinophilic gastrointestinal disease, and allergy to oats. For a more detailed list of inclusion and exclusion criteria, refer to the protocol in Appendix 1. Details on study design can be found in **Figure 1** and in the online **Supplementary Material**. Details on procedures, laboratory methods, and statistical analysis can also be found in the online **Supplementary Material**.

### Study approval

The Institutional Review Board of Stanford University School of Medicine approved the single site protocol. The NIH DAIT team and NIH DAIT DSMB were involved in reviewing the protocol and/or safety results. Written informed consent was obtained from parents or guardians of all study participants along with assent from participants of age 7 years and older. This trial is registered with c.

## Results

Forty cashew allergic participants were started on OIT and seen for their visits in a hospital setting (El Camino Hospital, Mt. View, CA). All 40 participants were included in the Intent-to-treat (ITT) population. The overall dropout rate by week 52 was 32.5% (13 out of 40 participants). Of the 13 dropouts, 1 dropped out due to abdominal discomfort. Other reasons for discontinuing study involvement included personal reasons, non-compliance, time constraints, dosing apprehension and COVID-19-related restrictions.

### Baseline demographic and clinical characteristics

Baseline clinical characteristics for the ITT population are summarized in **Table 1**. The median age at enrollment for cashew participants was 14 years (Inter Quartile Range (IQR) 11-15.3 years). More than two-thirds of participants had a history of comorbid conditions including asthma (47.5%), allergic rhinitis (70%), and atopic dermatitis (60%). The median cumulative tolerated dose (CTD) on screening DBPCFC was 13 mg (IQR 3–43 mg)and the median cashew-specific IgE was 13.1 IU/mL (IQR 4.0–53.5. All cashew allergic participants had demonstrated a systemic allergic reaction to the allergen for which they were treated in their medical history.

**Table 1:** Baseline demographic and clinical characteristics.

Baseline demographic and clinical characteristics	Cashew n = 40
Age (years) *	14 [11, 15.3]
Male	21 (52.5%)
Race	
White	11 (27.5%)
Asian	18 (45.0%)
Multiracial	9 (22.5)
Native Hawaiian or Pacific Islander	1 (2.5%)
Prefer not to report	1 (2.5%)
Hispanic or Latino	4 (10%)
Asthma and Allergy Condition Diagnosis	
Number of food allergies	6.5 [4, 10]
Asthma	19 (47.5%)
Allergic Rhinitis	28 (70%)
Atopic Dermatitis	24 (60%)
Baseline food challenge cumulative tolerated dose (CTD) (mg) *	13 [3, 43]
Baseline Total IgE (IU/mL) *	2209 [1173.3, 2560.8]
Specific IgE (IU/mL) *	13.1 [4.0, 53.5]
SPT Average wheal (mm) *	11.8 [8.9, 16.3]

Data are n (%) or median [IQR] *

### Time to maintenance

Most ITT participants (77.5%, 31/40) reached the 1000 mg maintenance dose of cashew allergen by a median of 209 days (95% CI: 205–260 days; **Supplementary Figure S1**). Given the constraints imposed by the COVID-19 pandemic, the actual duration of visits exceeded the originally planned study period. Consequently, we conducted a sensitivity analysis to determine maintenance times in relation to the initially designed study weeks. The median weeks to maintenance was about 182 days(26 weeks) (**Supplementary Figure S1**). There were no baseline demographic or clinical characteristics associated with time to maintenance (**Supplementary Figure S2**).

### Clinical outcomes

In the ITT population, 26 (65%) participants reached DS (i.e., no reaction or mild reaction on DBPCFC at 52 weeks) with CTD at least 2043 mg (**Table 2A**). At week 58, after 6 weeks of OIT avoidance, all 26 successfully met the SU endpoint (i.e., no reaction to mild reaction on DBPCFC at 58 weeks). DS and SU outcomes among the per protocol (PP) population are shown in **Table 2B**; 96% reached desensitization and all those who achieved desensitization reached sustained unresponsiveness). The participant who failed to achieve desensitization at Week 52 reached a CTD of 1043 mg of cashew. There were no baseline demographics or clinical characteristics associated with week 52 or week 58 clinical outcome success (**Supplementary Figure S3**).

**Table 2:** Clinical outcomes for desensitization and sustained unresponsiveness.

A. ITT		
		cashew n = 40
Desensitization	Success	26 (65%)
	Fail	14 (35%)
Sustained Unresponsiveness	Success	26 (65)%
	Fail	14 (35%)
B. PP		
		Cashew n = 27
Desensitization	Success	26 (96%)
	Fail	1 (4%)
Sustained Unresponsiveness		n = 26
	Success	26 (100%)
	Fail	0 (0%)

### Immunologic parameters

#### Plasma studies show a significant reduction in sIgE and an increase in sIgG4 for cashew

**Figure 3:**
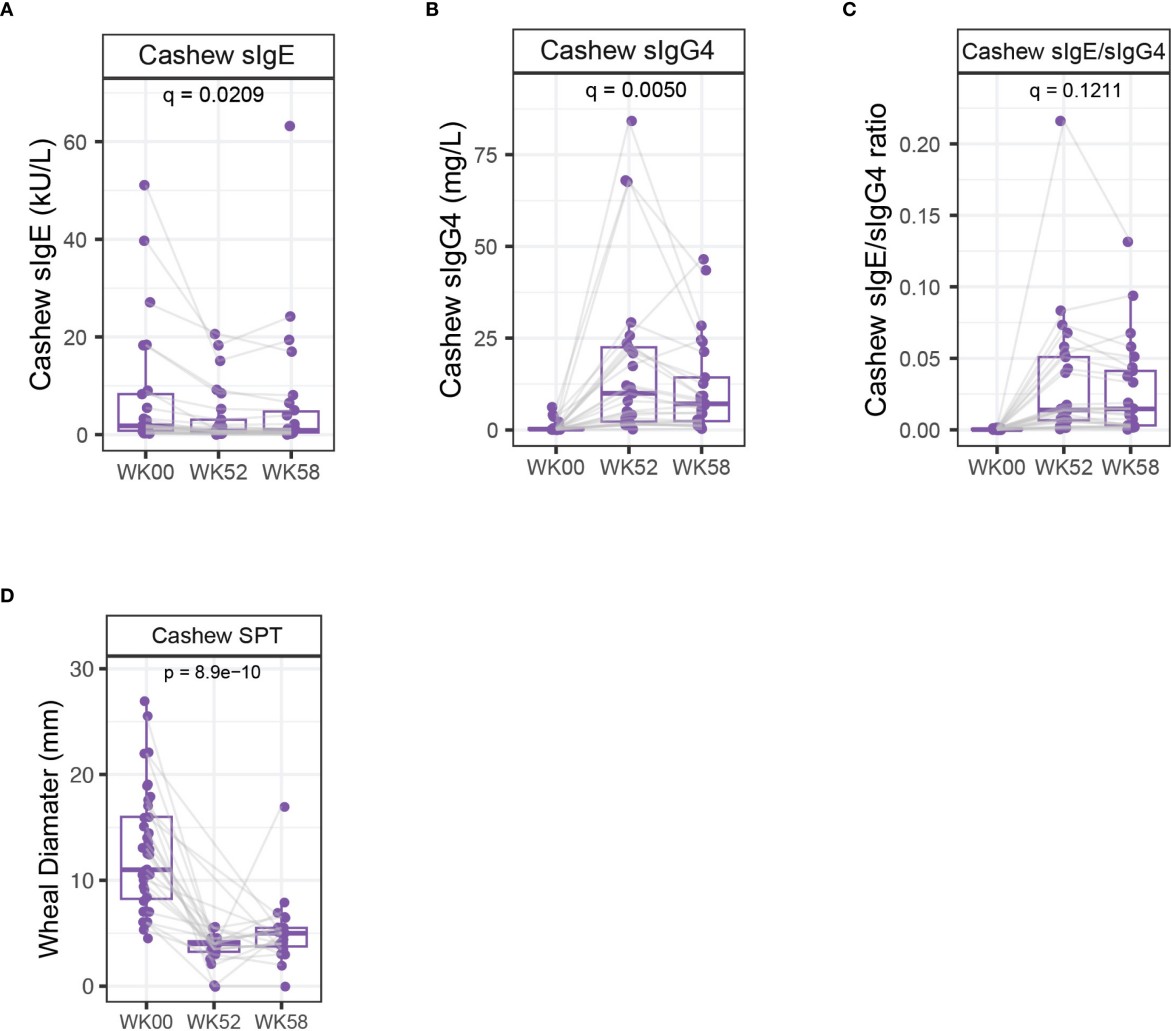
Serological markers and skin prick tests: Among the cashew population (*n* = 26). **(A)** sIgE **(B)** sIgG4 **(C)** Ratio of IgG4 to IgE at week 0 (Baseline), week 52 (post-OIT) and week 58 (post-avoidance). **(D)** SPT wheal diameter (mm) measurements across week 0, week 52 and week 58 (*n*: week 0 = 40, week 52 = 19, week 58 = 23). For boxplots, p-values were calculated using a repeated measures analysis of variance (ANOVA; adjusted for each sample and batch) to compare differences between groups. Grey lines represent 19 participants common across all three time points, which were used for repeated measures ANOVA. The boxplots depict the median, interquartile ranges, and range (whiskers), with outliers omitted to enhance the clarity of the Y-axis range visualization. p-values were adjusted for multiple hypothesis testing using false discovery rate (q-value).

Among cashew participants, there was a significant decrease in cashew sIgE (q=0.0209) and a significant increase in cashew sIgG4 (q=0.0050) at week 52 and week 58 compared to baseline while the ratio of cashew IgG4 to IgE remained relatively unchanged at weeks 52 and 58 (q=0.1211) (**Figures 3A–C**).

#### Cellular studies show downregulation of CRTH2 and low baseline expression of plasma TARC, EGF, and IP10 is predictive of tolerated dose post-avoidance

**Figure 4:**
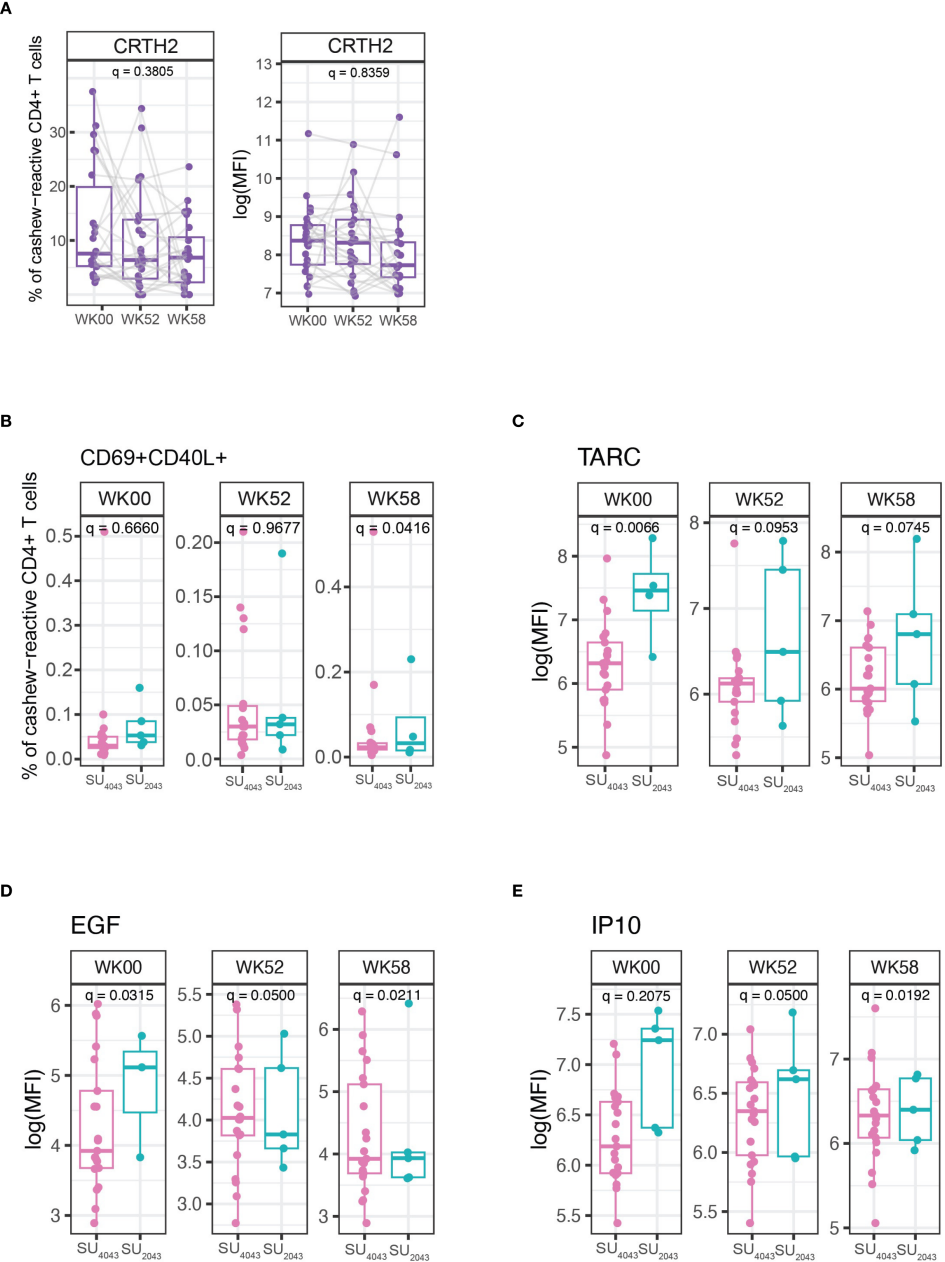
Frequency and mean fluorescent intensity (MFI). **(A)** CRTH2 + cashew-reactive CD4+ T cells at week 0 (Baseline), week 52 (post-OIT) and week 58 (post-avoidance) [q = 0.3805, p = 0.0485]. **(B)** Frequency of cashew-reactive (CD69^+^ CD40L^+^) CD4^+^ T cells among total CD4^+^ T cells at week 0, week 52 and week 58 between participants that achieved sustained unresponsiveness at a CTD of 4043mg (SU_4043_) and those who achieved sustained unresponsiveness at a CTD of 2043mg (SU_2043_)[week 0: q = 0.666, week 52: q = 0.9677, week 58: q = 0.0416]. Expression of **(C)** TARC [week 0: q = 0.0066, week 52: q = 0.0953, week 58: q = 0.0745] **(D)** EGF [week 0: q = 0.0315, week 52: q = 0.0500, week 58: q = 0.0211] and **(E)** IP10 [week 0: q = 0.2075, week 52: q = 0.0500, week 58: q = 0.0192] from plasma at week 0, week 52, and week 58 between SU_4043_ and SU_2043_ from cashew study participants that were assessed for cytokines and chemokines using Luminex. For the longitudinal analysis boxplots, p-values were calculated using a repeated measures analysis of variance (ANOVA; adjusted for each sample and batch) to compare differences between groups. For SU groups comparison boxplots, p-values were calculated using the *χ2* tests in mixed-effects models (Random effect: sample ID and batch) to compare differences between groups. The boxplots depict the median, interquartile ranges, and range (whiskers), with outliers omitted to enhance the clarity of the Y-axis range visualization. p-values were adjusted for multiple hypothesis testing using false discovery rate (q-value).

We employed multicolor flow cytometry to evaluate phenotypic changes in allergen-reactive (CD69^+^ CD40L^+^) CD4^+^ T cells post cashew allergen stimulation at baseline, week 52, and week 58. At week 52 and week 58, there was a decrease in cashew-reactive pathogenic Th2 CD4^+^CRTH2^+^ cells from baseline (q = 0.3805, p = 0.0485) (**Figure 4A**) (2). Manual gating was employed to identify allergen-reactive CD4^+^ T cell populations, with the gating strategy delineated in **Supplementary Figure S4A**. Analysis revealed no statistically significant differences in the frequencies of CD69^+^CD40L^+^ allergen-reactive CD4^+^ at week 52 and week 58 compared to baseline in the cashew participants (**Supplementary Figure S4B**).

In parallel with the CD69/CD40L upregulation assay performed on allergen reactive CD4+ T cells, we also used cashew-epitope specific tetramers to immunophenotype cashew -specific CD4^+^ T cells among unstimulated PBMCs from 3 HLA-compatible participants (**Supplementary Table S1**). Manual gating was utilized to identify cashew-specific (tetramer^+^) CD4^+^ T cell populations. The detailed gating strategy is illustrated in **Supplementary Figure S5B**. The majority (> 85%) of tetramer^+^ CD4^+^ T cells exhibited memory (CD45RA^-^) phenotype with Th2-A polarization (CRTH2^+^ CXCR3^-^ CXCR5^-^) at baseline. The median frequency of cashew tetramer^+^ CD4^+^ T cells at baseline was 0.96%, and there was a trend in decrease (q=0.1941, p=0.1941) in this frequency at weeks 52 and 58 (**Supplementary Figure S5B**) to 0.41% and 0.21%, respectively. We also observed a slight increase in the frequency of CCR6^+^ tetramer^+^ CD4^+^ T cells (q=0.8716, p=0.1712) at weeks 52 and 58, though this was not statistically significant. (**Supplementary Figure S5C**).

Following the period of avoidance, two threshold levels of SU were further differentiated based on the CTD in the DBPCFC. Participants with a DBPCFC of CTD of 2043 mg were classified as SU_2043_ and those who obtained a CTD of 4043 mg during their DBPCFC were classified as SU_4043_. These comparisons could potentially further identify unique immunophenotypes associated with SU clinical outcomes (in terms of higher or lower dose achieved on DBPCFC). On comparing the flow cytometry data between SU_4043_ and SU_2043_ for cashew-allergic participants, we observed that SU_4043_ participants had a lower frequency of allergen-reactive CD4^+^ T cells than SU_2043_ at week 58 (p = 0.04, **Figure 4B**).

### Plasma and PBMC supernatant proteomics

In addition to the CD4^+^ T cell phenotype, we investigated OIT-induced changes in plasma and PBMC culture supernatant composition using Luminex. The comparison of cytokine and chemokine expression in the plasma of SU_4043_ *vs*. SU_2043_ participants revealed a significantly higher baseline expression of pro-inflammatory cytokines TARC/CCL17, EGF, and IP10 in SU_2043_ participants (**Figures 4C–E**). The Th2 (IL-4, IL-5, IL-9, IL-13), Th1 (IFN-γ), and regulatory cytokine IL-10 profiles were evaluated at week 0, week 52, and week 58 through Luminex using plasma samples (**Supplementary Figure S6**) and allergen-stimulated culture supernatants (**Supplementary Figure S7**). However, no statistically significant changes in the expression of these cytokines were detected at week 52 and week 58 compared to baseline.

### Skin tests

In this study, we quantified skin prick test (SPT) wheal sizes among participants undergoing DBPCFCs at week 0, week 52 and week 58. Among cashew participants, the median SPT wheal diameter observed was 11.8mm at week 0, 4mm at week 52 and 5mm at week 58. Statistical analysis revealed a significant decrease in wheal diameter (p=8.9E-10) participants at weeks 52 and 58 compared to baseline measurements (**Figure 3D**).

### Safety

Across all groups, the number of OIT-induced adverse events (AE) and the percentage of participants with AEs decreased by study phase (**Supplementary Table S2**, **Supplementary Table S3**). Total 40 participants experienced 713 AEs; the most common AEs during were gastrointestinal (26/40, 65%), followed by those affecting the skin (10/40, 25%). There were no baseline characteristics associated with AE rates during the study (**Supplementary Figure S8**). Three participants reported 4 epinephrine injections for AEs that affected gastrointestinal and skin; all events resolved within a few minutes with no sequelae (**Supplementary Table S4**). No episodes of hypoxia or neurological compromise occurred. There were no OIT-related serious AEs. Two AEs met the anaphylaxis criteria [see protocol, Appendix 1]; both were resolved after one dose of injectable epinephrine. One participant reported an episode of AEs related to accidental ingestion of OIT food allergen (**Supplementary Table S5**).

### Food allergy quality of life questionnaire

Mixed effect models were used to analyze the Food Allergy Quality of Life Questionnaire (FAQLQ) scores from week 0 to week 52 to week 58. Median FAQLQ Adult Form scores increased from 2.4 at baseline (n=1) to 3.0 at week 58 (n=2) (p=0.002) The FAQLQ Parental Burden score significantly improved over time, decreasing from 1.4 at baseline (n=27) to 0.9 at week 58 (n=15) (p=0.03), meeting the Minimal Important Difference (MID) threshold of 0.5 for this questionnaire, indicating a clinically meaningful improvement (3, 4). No statistically significant change was observed for other FAQLQ scores, which may be due to the small sample size, limiting the study’s power to detect meaningful changes (**Supplementary Figure S9**, **Supplementary Table S6**).

## Discussion

The MOTIF (T Cell Reagent Research for Monitoring T Cells in Food Allergy, NCT 03504774) study results demonstrate that using OIT to treat cashew food allergy has a high likelihood of achieving DS and SU among allergic participants. Due to the size of our cohort and age range between children and adults (40 cashew-allergic participants), our findings could be relevant for DS practices. At week 52, 26 (65%) of the cashew-allergic participants, of the ITT population, passed the food challenge. During the SU determination at week 58, 26 (65%) of the cashew-allergic participants of the ITT population, passed the DBPCFC. This level of success in SU was likely due to the short period of withdrawal (6 weeks). The high rates of DS and SU observed in our study suggest that cashew OIT are effective and safe for treating IgE-mediated cashew allergy in patients, adding to the literature for these allergens.

In addition to evaluating the efficacy of cashew OIT, we also aimed to investigate the mechanistic basis of allergen-specific T-cell responses to identify potential therapeutic targets. Allergen-specific type 2 helper T (Th2) cells play a central role in the allergic inflammatory pathway. Pathogenic Th2A and Th2 cells are characterized by the expression of CRTH2 (2, 5). In our study, using immunophenotyping by the CD69 CD40L upregulation assay on a subset of allergic patients, we demonstrated a decrease in the frequency of such CRTH2^+^ pathogenic effector Th2 cells to a modest extent at Week 52 and significantly by Week 58 compared to baseline indicating an immune downregulation of the Th2A phenotype as evidenced by decreased CRTH2 expression. As demonstrated by Wambre et al. (2017), successful peanut OIT leads to a decrease in the frequency and expression of the Th2A phenotype, which is characterized by high levels of CRTH2.This suggests that OIT induced a similar shift from a Th2A-dominated allergic phenotype towards a Th1/Th17-like profile correlating with the induction of DS and SU (6–9). In addition to using allergen-specific tetramers, we have also initiated efforts to explore the use of spheromers for the identification of allergen-specific CD4^+^ T cells. Spheromers, as described by Vamsee et al., are nanoparticles designed to present multiple copies of immunodominant peptides on their surface, potentially offering improved binding avidity compared to tetrameric structures (10). Further optimization of spheromers for cashew immunodominant peptides is currently underway.

Elevated levels of IP10 (Interferon-gamma-inducible protein 10) have been previously associated with airway inflammation in asthma (11). In our study, SU_2043_ participants had higher baseline levels of IP10 compared to SU_4043_. This observation aligns with the higher prevalence of asthma (60%) in the SU_2043_ group compared to the SU_4043_ group (31.6%). Increased levels of TARC (Thymus and Activation-Regulated Chemokine) and EGF (Epidermal Growth Factor) have been linked to the pathogenesis of atopic dermatitis, a condition mediated by preferential Th2 recruitment (12, 13). Our analysis found higher baseline levels of TARC and EGF in the SU_2043_ group, which also had a higher prevalence of atopic dermatitis (80%) compared to the SU_4043_ group (42.1%). The higher baseline levels of IP10, TARC, and EGF in the SU_2043_ group, coupled with the higher prevalence of asthma and atopic dermatitis, suggest that these comorbid conditions and associated biomarkers may reduce the likelihood of achieving SU. Our novel findings indicate that baseline levels of IP10, TARC, and EGF may have potential as predictive biomarkers for enhanced OIT success. Participants with lower levels of these biomarkers at baseline may be more likely to achieve SU at higher doses of allergen challenge, potentially leading to better long-term outcomes. It is important to note that while continued dosing during OIT may desensitize participants, those with higher baseline levels of these biomarkers and a history of asthma or atopic dermatitis may not be able to achieve tolerance. In such cases, continued dosing at lower levels may be more appropriate, as avoiding allergen exposure altogether is likely not protective.

These findings highlight the potential value of baseline biomarker assessment in predicting OIT outcomes and tailoring treatment strategies for individual patients based on their comorbidities and biomarker profiles. Our study demonstrates success in achieving DS and SU in those with cashew allergy and addresses a notable knowledge gap. The substantial representation of cashew-allergic participants bolsters the credibility and applicability of our findings and enhances understanding of this understudied allergen.

The inclusion of immunophenotyping enhanced our understanding of the immunological mechanisms underlying oral immunotherapy (OIT). Identifying potential predictive markers associated with co-morbidities broadens the potential clinical relevance. Despite acknowledged limitations including the open-label design, relatively short follow-up, the higher than expected dropout rate (mostly due to time constraints and COVID-19-related restrictions), and the absence of blood samples for participants who withdrew before DS and SU food challenges, our study lays a foundation for advancing OIT research and its potential applications in managing cashew food allergies.

## Data Availability

The raw data supporting the conclusions of this article will be made available by the authors, without undue reservation.
