# Supplementary material for: Trends in allergen-reactive CRTH2+ T cells and TARC associated with successful outcomes in a phase 2 cashew oral immunotherapy study
Source: Front Immunol. 2025 Oct 22;16:1655975. doi: 10.3389/fimmu.2025.1655975 (PMC12586961; doi:10.3389/fimmu.2025.1655975)
Supplement: Supplementary file 1 [file DataSheet1.docx]

**SUPPLEMENTARY MATERIALS**

**METHODS**

**Procedures**

All participants were screened using published, standardized procedures of food challenges, food-allergen (FA)-specific IgE, and skin prick test, as detailed in the protocol. Food challenges were conducted in accordance with PRACTALL consensus guidelines.[1] No more than 40 weeks could elapse between the qualifying DBPCFC and the initial dosing day.

Safety parameters and compliance were monitored throughout the study using the CoFAR grading scale for systemic allergic reactions version 1.0 and Common Terminology Criteria for Adverse Events (CTCAE) version 5.0 for non-allergic reactions using daily diaries and in-clinic research unit responses. Full details of safety monitoring and reporting are in the protocol (appendix 1). Frequent instruction was given by trained personnel on the use of reaction medications and dosing. Participants had access to trained research clinicians via a 24-hour emergency call line throughout the study.

The study procedure is depicted in Figure 1. All enrolled participants underwent OIT starting at 5 mg allergen, with dose escalation every two weeks to reach a maintenance dose of 1,000 mg at week 28, which they maintained for 24 weeks (up to Week 52). At week 52, participants underwent DBPCFC to assess for desensitization. Participants who passed their food challenge at week 52 with no reaction or mild objective reactions to up to a cumulative tolerated dose of 2043 mg of the FA in their OIT were considered desensitized and therefore, had successfully met the study’s primary endpoint. Desensitized participants then continued in the study and underwent a withdrawal from OIT for 6 weeks to examine mechanisms underlying sustained responsiveness (SU); SU was defined as a participant passing a DBPCFC with no reaction or mild objective reaction to up to a cumulative 2043 mg of the FA in their OIT at week 58. The highest dose that participants can ingest was 4043 mg at week 52 and week 58 food challenges.

Participants and caregivers were asked to fill out the food allergy quality of life questionnaires (FAQLQ) questionnaires at baseline, week 52, and week 58. FAQLQ are validated questionnaires designed separately for 0-12, 13-17, and 18+ year old food allergic patients.[2]

**Laboratory methods**

**Blood draws and processing**

Blood was collected from the study participants undergoing OIT by venipuncture at week 0 (baseline), week 52, and week 58. Standardized procedures were used to isolate plasma and peripheral blood mononuclear cells (PBMCs) from the blood by Ficoll-based density gradient centrifugation. Aliquots of plasma and PBMCs were stored at -80°C and in liquid nitrogen, respectively, until they were thawed for testing [3].

**Serology**

Cashew-specific IgE and IgG4 plasma levels for the respective allergen-treated study participants were measured by ImmunoCAP fluorescence enzyme immunoassay (Phadia Immunology Reference Laboratory/Thermo Fisher Scientific). Upper and lower detection limits for specific IgE were 100 kUA/L and 0.1 kUA/L, respectively and these were replaced with 101 kUA/L and 0.01 kUA/L. Upper and lower detection limits for specific IgG4 were 30 mgA/L and 0.1 mgA/L, respectively, and these were replaced with 31 mgA/L and 0.01 mgA/L.

**Flow Cytometry-CD69, CD40L upregulation assay**

Baseline, week 52, and week 58 PBMCs from 24 cashew-treated participants were thawed and re-suspended in RPMI with 5% heat-inactivated human AB serum and Penicillin-Streptomycin at a concentration of 3x10^6^ cells/mL in polypropylene FACS tubes. The PBMCs were rested overnight at 37^o^C and 5% CO_2_ followed by stimulation with 200 μg/mL of cognate allergen (i.e., cashew ) protein solution for 24 hours. Cashew solutions used for *ex vivo* stimulation were derived from the flours used for DBPCFC in the clinic, dissolved in PBS and sterilized by filtration as previously described.[4] Unstimulated PBMCs served as controls. Brefeldin A (5 μg/mL; Biolegend, San Diego, CA) and Monensin (2 μM, Biolegend, San Diego, CA) were added to allergen-stimulated as well as unstimulated PBMCs for the last 4 hours of incubation to inhibit vesicular transport of CD40L and CD69.

At the end of the 24-hour incubation, 120 μL of PBMC culture supernatant was collected for Luminex assay and PBMCs were transferred into a 96-well, V-bottom plate. Harvested PBMCs were washed with CyFACS buffer (Dulbecco’s PBS + 0.1% BSA+ 0.2 M EDTA + 0.1% Sodium Azide) and stained with a 21-marker flow antibody panel (Table S7). Data were acquired using BD Symphony A5^™^ cytometer and analyzed by manual gating. The gating scheme is illustrated in Figure S7. Live CD4^+^ cells overexpressing CD69 and CD40L in response to allergen stimulation were identified as allergen-reactive.

**Tetramer assembly and staining**

A total of 5 cashew-component (Ana o 1, Ana o 2) epitope-specific and 5 shrimp-component (Pen m 2, Pen m 1) epitope-specific tetramers were custom assembled (Table S1)^4^. A total of 10 participants were found to be HLA-compatible with the available pool of tetramers, of which 5 (3 cashew, 2 shrimp) participants showed detectable tetramer^+^ staining. Thawed PBMCs were treated with 50 nM dasatinib at 37^o^C for 10 min followed by staining with cognate tetramer for 2 hours at room temperature. Tetramer+ PBMCs were enriched using PE beads. Enriched cells were stained with the panel of surface antibodies as described above.

**Luminex**

PBMC culture supernatant samples collected as described above were centrifuged at 10,000g for 10 minutes at 4°C to remove cellular debris. Post-centrifugation, 100μLs of the culture supernatant was transferred into a sterile, labeled microfuge tube and stored at -80°C until the Luminex assay was performed. The culture supernatants were probed using a 48-plex Cytokine/Chemokine Magnetic Bead Panel (HCYTA-60K-PX48, Millipore Sigma, Burlington, MA). Plasma aliquots were probed with the following Luminex panels: HCYTA-60K-PX48, HCP2MAG-62K-PX23, HSP1MAG-63K-06, and HADCYMAG-61K-03 (Millipore Sigma).

**Statistical analysis**

Sample size considerations can be found in Appendix 1. The intent-to-treat (ITT) principle was defined as all enrolled participants; the per-protocol (PP) population was defined as only individuals who reached each challenge endpoint. The clinical efficacy analyses were primarily performed in the ITT population, and further summarized in PP population as secondary analysis. We tested the association between each baseline characteristic and desensitization and SU using logistic regression. The time to reach 1,000 mg of maintenance was illustrated descriptively using the Kaplan-Meier plot. Participants who dropped out of the study were censored at their dropout date. The associations between baseline characteristics and time to reach maintenance were assessed using Cox Proportional Hazard regression. The OIT-related adverse events (AE) and percentages of participants with any AEs were presented descriptively. The AE rate per participant was calculated by the number of AEs divided by the total number of doses. Linear mixed effect models ^5^were fit to determine whether baseline characteristics were associated with AE rate from baseline to week 52. The mean total scores and the mean domain scores were calculated for each participant or caregiver at baseline and post-study for FAQLQ. The changes in scores from baseline to week 58 were assessed using the linear mixed effect model. All analyses were conducted using two-sided tests where p<0.05 was determined to be the cut-off for statistical significance.

All mechanistic and serological analyses were performed using mixed-effect models and repeated measure ANOVA. Repeated measures over three time points were compared to assess changes in biomarkers over time using repeated measure ANOVA, adjusted for sample ID and batch. Pairwise analyses between any two-time points (week 0 vs week 52, week 0 vs week 58 and week 52 vs week 58) or groups (SU_4043_ vs SU_2043_) were performed using the *χ*^2^ test in mixed effects models where time was used as a fixed effect and the sample ID and batch were used as random effects, using lmerTest R package[5]. p-values were adjusted for multiple hypothesis testing (q-value) using false discovery rate (FDR) q-value. Markers with p-value <0.05 or q-value <0.1 were considered significant. All statistical analyses were performed using R software (version 4.2.3). All the statistical plots were created using ggplot2 package in R (Version 3.4.4)

**References**

1. Sampson, H.A., et al., *Standardizing double-blind, placebo-controlled oral food challenges: American Academy of Allergy, Asthma & Immunology-European Academy of Allergy and Clinical Immunology PRACTALL consensus report.* J Allergy Clin Immunol, 2012. **130**(6): p. 1260-74.

2. Flokstra-de Blok, B.M.J., *Food Allergy Quality of Life Questionnaires (FAQLQ)*, in *Encyclopedia of Quality of Life and Well-Being Research*, A.C. Michalos, Editor. 2014, Springer Netherlands: Dordrecht. p. 2319-2322.

3. Fuss, I.J., et al., *Isolation of whole mononuclear cells from peripheral blood and cord blood.* Curr Protoc Immunol, 2009. **Chapter 7**: p. Unit7.1.

4. Zhou, X., et al., *A positive feedback loop reinforces the allergic immune response in human peanut allergy.* J Exp Med, 2021. **218**(7).

5. Kuznetsova, A., P.B. Brockhoff, and R.H.B. Christensen, *lmerTest package: tests in linear mixed effects models.* Journal of statistical software, 2017. **82**(13).

**Table S1** Cashew- and Shrimp-specific Tetramers

| **PEPTIDE/POSITION** | **ALLELE** | **SEQUENCE** | **PPID** |
| --- | --- | --- | --- |
| **Cashew tetramers** |  |  |  |
| Ana o2  289-308 | DRB1*04:04 | PARADIYTPEVGRLTTLNSL | MTF031, MTF050 |
| Ana o2  321-340 | DRB1*15:01 | EKGVLYKNALVLPHWNLNSH | MTF005 |

**Table S2**. OIT-induced adverse events.

|  | **Overall** | **Build up** | **Maintenance** |
| --- | --- | --- | --- |
| **Number of events** | 713 | 695 | 18 |
| Category |  |  |  |
| Gastrointestinal | 234 (33%) | 228 (33%) | 6 (33%) |
| Skin | 29 (4%) | 20 (3%) | 9 (50%) |
| Respiratory | 11 (2%) | 11 (2%) | 0 (0%) |
| General | 78 (11%) | 78 (11%) | 0 (0%) |
| Epinephrine | 4 (1%) | 3 (0.4%) | 1 (6%) |
| Concomitant medication | 117 (16%) | 113 (16%) | 4 (22%) |
| Emergency room visit | 2 (0.3%) | 1 (0%) | 1 (6%) |
| Anaphylaxis | 2 (0.3%) | 2 (0.3%) | 0 (0%) |
| CoFAR grading |  |  |  |
| Grade 1 | 644 (90%) | 627 (90%) | 17 (94%) |
| Grade 2 | 69 (10%) | 68 (10%) | 1 (6%) |

**Table S3**. Participants who had any OIT-induced adverse events.

|  | **Build up** | **Maintenance** |
| --- | --- | --- |
| Number of participants | 40 | 31 |
| Any adverse event***** | 40 (100%) | 6 (19%) |
| Category |  |  |
| Gastrointestinal | 26 (65%) | 2 (7%) |
| Skin | 10 (25%) | 4 (13%) |
| Respiratory | 5 (13%) | 0 (0%) |
| General | 8 (20%) | 0 (0%) |
| Epinephrine | 2 (5%) | 1 (3%) |
| Concomitant medication | 25 (63%) | 3 (10%) |
| Emergency room visit | 1 (3%) | 1 (3%) |
| Anaphylaxis | 2 (5%) | 0 (0%) |
| Maximum CoFAR grading** |  |  |
| Grade 1 | 20 (50%) | 5 (16%) |
| Grade 2 | 20 (50%) | 1 (3%) |

*Number of participants with at least one adverse event out of the number of participants at each phase.

**Maximum CoFAR grading for participants with at least one adverse event out of the number of enrolled participants

**Table S4.** Description of injectable epinephrine use related to OIT dosing.

| **Participant** | **Allergen** | **Phase** | **AE term** | **CoFAR Grade** | **Epi Dose Given** | **AE Outcome** |
| --- | --- | --- | --- | --- | --- | --- |
| 1 | Cashew | Build up | Nausea | Grade 2 | 1 | Resolved without sequelae |
| 2 | Cashew | Maintenance | Urticaria | Grade 2 | 1 | Resolved without sequelae |
| 3 | Cashew | Build up | Vomiting | Grade 2 | 1 | Resolved without sequelae |
|  |  | Build up | Nausea | Grade 2 | 1 | Resolved without sequelae |

GI: gastrointestinal; Epi: epinephrine

**Table S5**. Description of accidental ingestion per study phase.

| **Participant** | **Allergen** | **Phase** | **AE term** | **CoFAR Grade** | **Due to OIT Food Allergen (FA)** |
| --- | --- | --- | --- | --- | --- |
| 1 | Cashew | Maintenance | Abdominal pain | Grade 2 | OIT FA |

**Table S6**. Food Allergy Quality of Life Questionnaire (FAQLQ) Scores

|  | **Week 0** | **Week 52** | **Week 58** | **p-value (week 0 to week 58)** |
| --- | --- | --- | --- | --- |
| **FAQLQ Age 0-12** | n=18 | n=8 | n=8 |  |
| **Median (IQR)** |  |  |  |  |
| **Total** | 2.5 (1.8, 3.3) | 1.6 (1.0, 2.5) | 1.9 (1.4, 3.1) | 0.4 |
| **Emotional Impact** | 1.8 (1.4, 3.1) | 1.1 (0.7, 2.7) | 1.8 (1.5, 2.8) | 0.6 |
| **Food Anxiety** | 3.1 (2.0, 3.9) | 1.8 (1.3, 2.7) | 2.4 (1.6, 3.9) | 0.5 |
| **Social Limitation** | 2 (1.2, 3.1) | 1 (0.7, 2.2) | 1.1 (0.8, 2.0) | 0.5 |
| **FAQLQ Age 13-17** | n=13 | n=5 | n=5 |  |
| **Median (IQR)** | 2.3 (2.0, 3.9) | 1.9 (1.5, 2.7) | 1.8 (1.7, 2.8) | 0.2 |
| **FAQLQ Adult Form** | n=1 | n=3 | n=2 |  |
| **Median (IQR)** | 2.4 (2.4, 2.4) | 2.7 (2.1, 2.9) | 3.0 (2.9, 3.2) | NA* |
| **FAQLQ Parental Burden** | n=27 | n=15 | n=15 |  |
| **Median (IQR)** | 1.4 (0.5, 2.4) | 0.8 (0.2, 1.2) | 0.9 (0.5, 1.9) | 0.03 |

* Due to the small sample size (n=1 at Week 0 and n=2 at Week 58), statistical analysis was not performed for the FAQLQ Adult Form.

**Table S7** Flow Cytometry antibody panel

| **#** | **Serial No.** | **Marker** | **Manufacturer & Catalog#** | **Clone** | **Isotype** |
| --- | --- | --- | --- | --- | --- |
| 1 | BB515 | CD25 | BD 565096 | M-A251 | Mouse BALB/c IgG1, κ |
| 2 | PerCPCy5.5 | CD23 | Biolegend 338518 | EBVCS-5 | Mouse IgG1, κ |
| 3 | PE | CD40L/CD154 | Biolegend 310806 | 24-31 | Mouse IgG1, κ |
| 4 | PE-CF594 | CCR4 | BD 565391 | 1G1 | Mouse C57BL/6 IgG1, κ |
| 5 | PE-Cy7 | CRTH2 | Biolegend 350118 | BM16 | Rat IgG2a, κ |
| 6 | APC | GPR15 | Biolegend 373006 | SA302A10 | Mouse IgG2a, κ |
| 7 | A700 | ST2 | R&D FAB5232N-100 | 2154B | recombinant human ST-2/ IL-33R |
| 8 | AF780 | CD127 | eBio 47-1278-42 | eBioRDR5 | Mouse / IgG1, kappa |
| 9 | BV421 | IL10R | BD 742942 | 3F9 | Rat F344, also known as Fischer, CDF IgG2a, κ |
| 10 | BV480 | CD38 | BD 566137 | HIT2 | Mouse IgG1, κ |
| 11 | BV570 | CD45RA | Biolegend 304132 | HI100 | Mouse IgG2b, κ |
| 12 | BV605 | CD3 | Biolegend 300460 | UCHT1 | Mouse IgG1, κ |
| 13 | BV650 | CXCR3 | Biolegend 353730 | G025H7 | Mouse IgG1, κ |
| 14 | BV711 | CD27 | BD 564893 | M-T271 | Mouse BALB/c IgG1, κ |
| 15 | BV750 | PD1 | BD 747446 | EH12.1 | Mouse IgG1, κ |
| 16 | BV786 | CCR6 | Biolegend 353422 | G034E3 | Mouse IgG2b, κ |
| 17 | BUV395 | CXCR5 | BD 740266 | RF8B2 | Rat LOU, also known as Louvain,LOU/C, LOU/M IgG2b, κ |
| 18 | BUV496 | CD8 | BD 612942 | RPA-T8 | Mouse IgG1,κ//CD8 |
| 19 | BUV496 | CD14 | BD 750381 | M5E2 | Mouse IgG2a,κ//CD14 |
| 20 | BUV496 | CD19 | BD 612938 | SJ25C1 | Mouse BALB/c IgG1, κ// CD19 |
| 21 | BUV563 | CD161 | BD 749223 | HP-3G10 | Mouse BALB/c IgG1, κ |
| 22 | BUV661 | CD69 | BD 750213 | FN50 | Mouse IgG1, κ |
| 23 | BUV737 | CD28 | BD 612815 | CD28.2 | Mouse C3H x BALB/c IgG1, κ |
| 24 | BUV805 | CD4 | BD 612887 | SK3 | Mouse BALB/c IgG1, κ |

**Figure S1:**  Kaplan-Meier plot for time from OIT started to reach 1000 mg maintenance


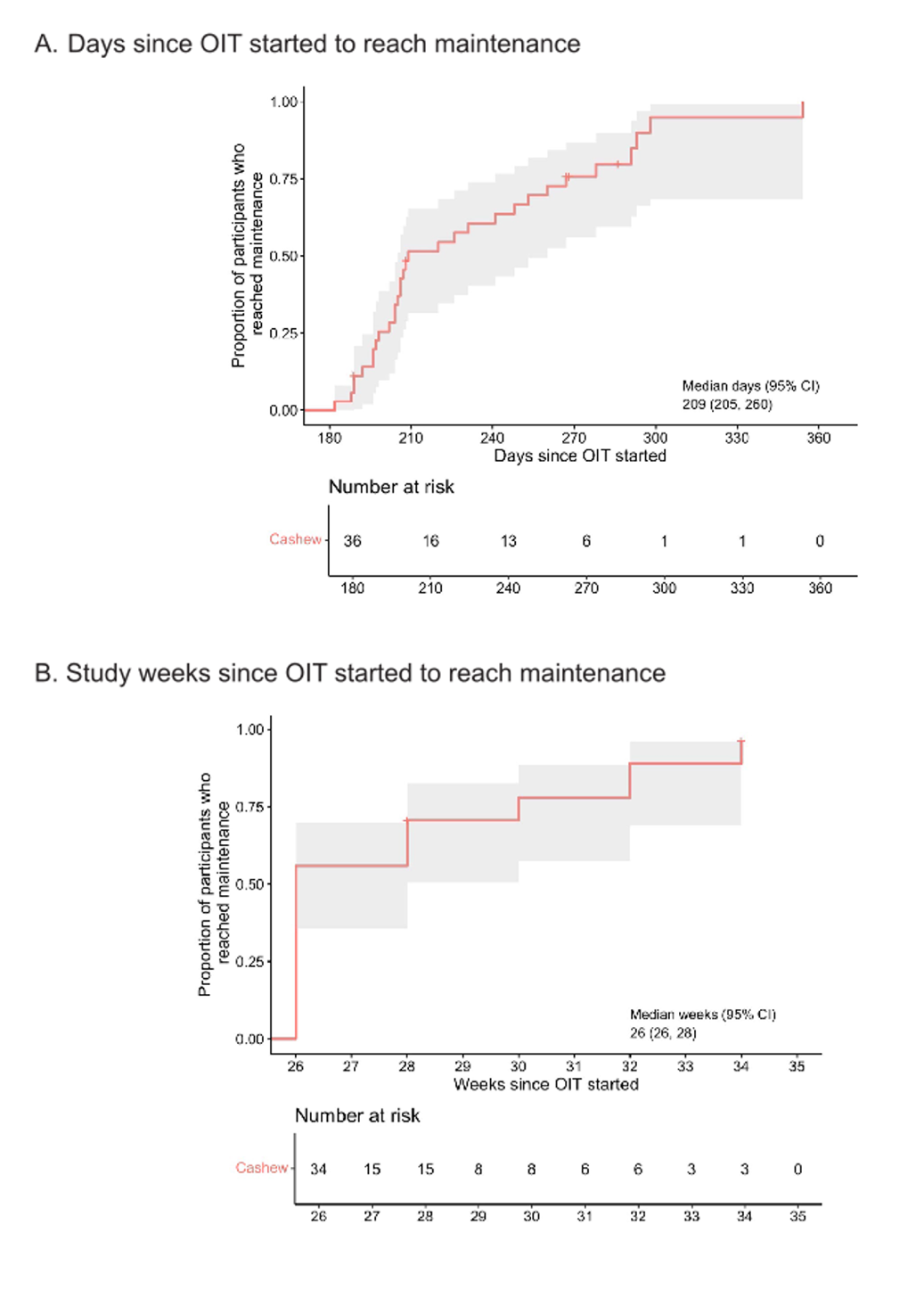


**Figure S1:** Study days (A) and weeks (B) since OIT started to reach 1000 mg maintenance. Participants who dropped out of the study were censored at their dropout date. Bands correspond to the 95% confidence intervals.

**Figure S2:** Associations between baseline characteristics and days to reach maintenance.


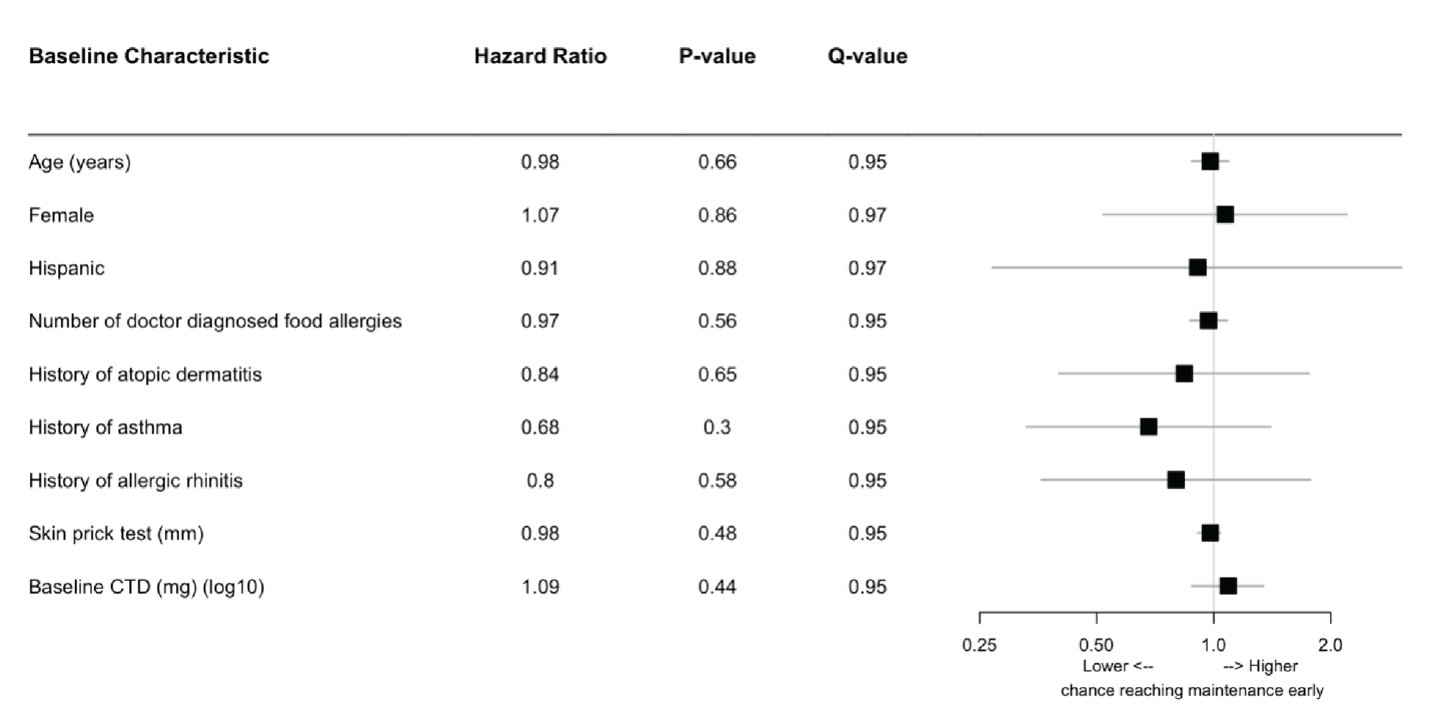


**Figure S2**: Associations between baseline characteristics and days to reach maintenance in ITT analysis cohort. Each characteristic was fit to a Cox proportional hazard regression model. An estimate above 1 (gray line) denotes an association with higher chance of reaching maintenance early, while an estimate below 1 denotes an association with less. Q-value is the FDR-adjusted P-value.

**Figure S3:**  Associations between baseline characteristics and DBPCFC success at weeks 52 and 58.

**
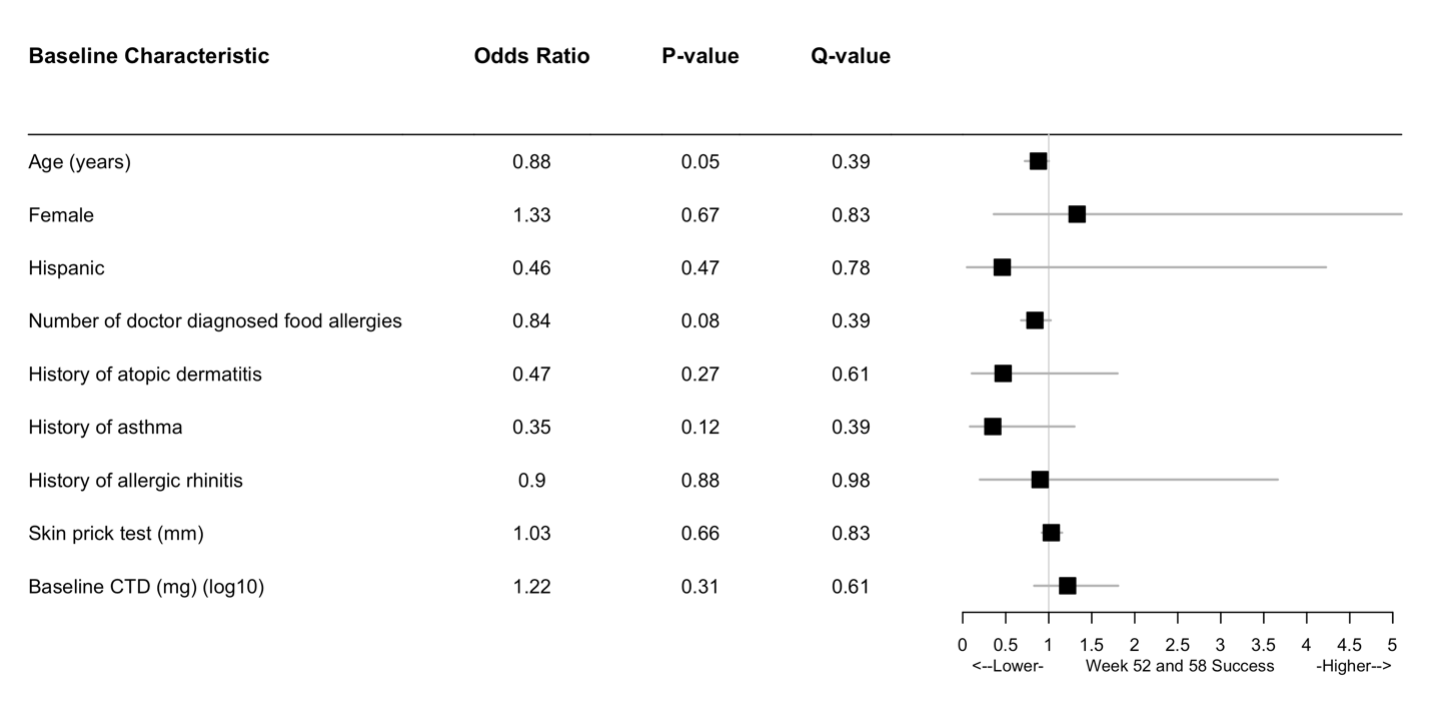
 Figure S3:**

Associations between baseline characteristics and DBPCFC success in the ITT analysis cohort at weeks 52 and 58. The same group of participants passed the DBPCFCs at both time points. Each characteristic was analyzed using a logistic regression model. An estimate above 1 (indicated by the gray line) represents a positive association with a higher likelihood of passing the DBPCFC at the cumulative tolerated dose (CTD) of 2043 mg, while an estimate below 1 indicates a negative association. Q-values represent the FDR-adjusted P-values.

***
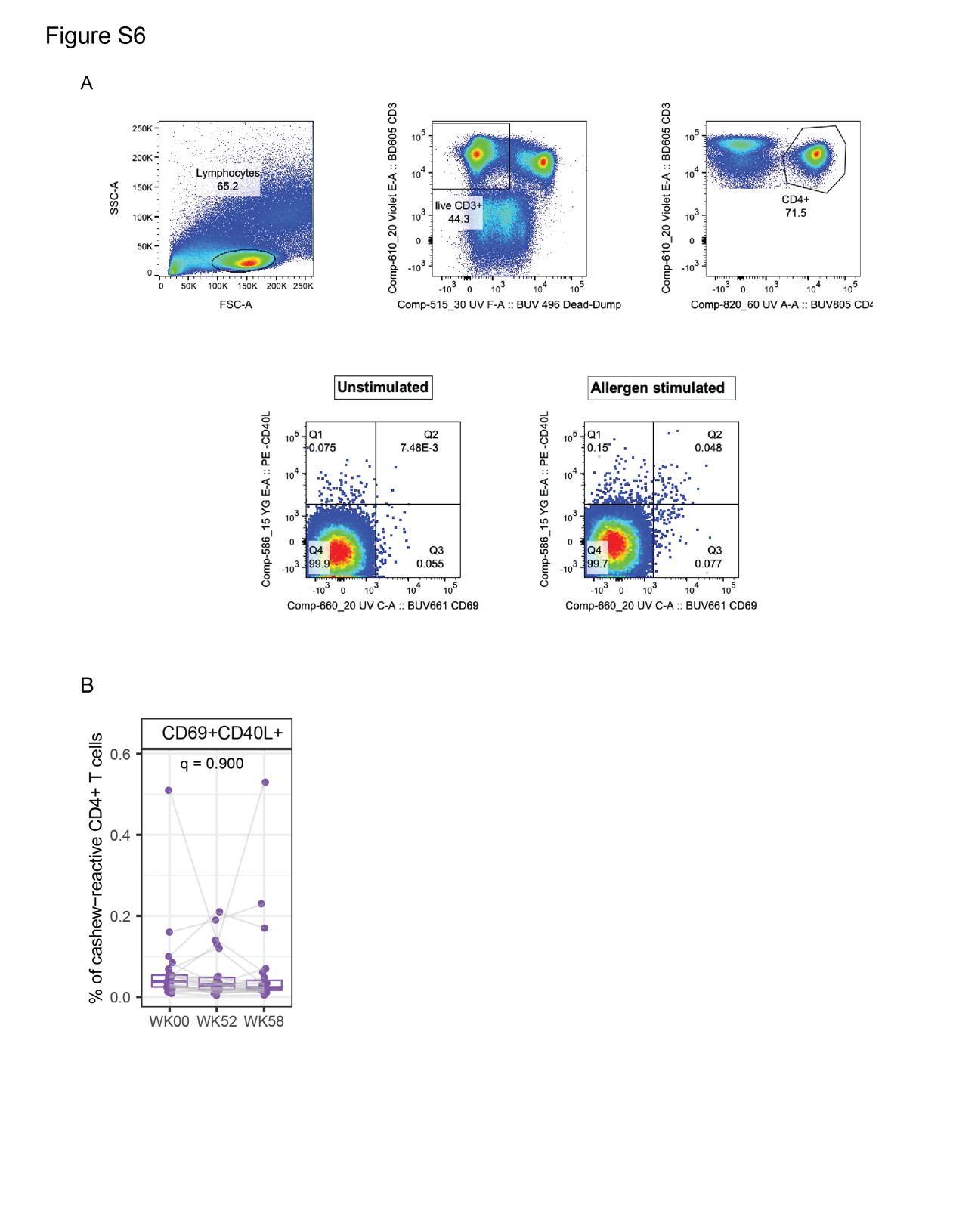
***F***igure S4:*** Allergen-Reactive CD4+ T Cells Identification and Frequency

***Figure S4***: (A) Gating scheme for identification of allergen-reactive CD4^+^ (CD69^+^ CD40L^+^) T cells on *ex vivo* allergen stimulation of PBMCs and allergen-reactive CD4^+^ cells among unstimulated and allergen-stimulated PBMCs. (B) Frequency of cashew-reactive(CD69^+^ CD40L^+^) CD4^+^ T cells among total CD4^+^ T cells at Week 0 (Baseline), Week 52 (post-OIT), and Week 58 (post-avoidance). For boxplots, p-values were calculated using a repeated measures analysis of variance (ANOVA; adjusted for each sample and batch) to compare differences between groups. The boxplots depict the median, interquartile ranges, and range (whiskers), with outliers omitted to enhance the clarity of the Y-axis range visualization. p-values were adjusted for multiple hypothesis testing using false discovery rate (q-value).

**Figure S5**: Identification and Frequency of Allergen-Specific CD4+ T Cells using allergen-specific tetramers.

***
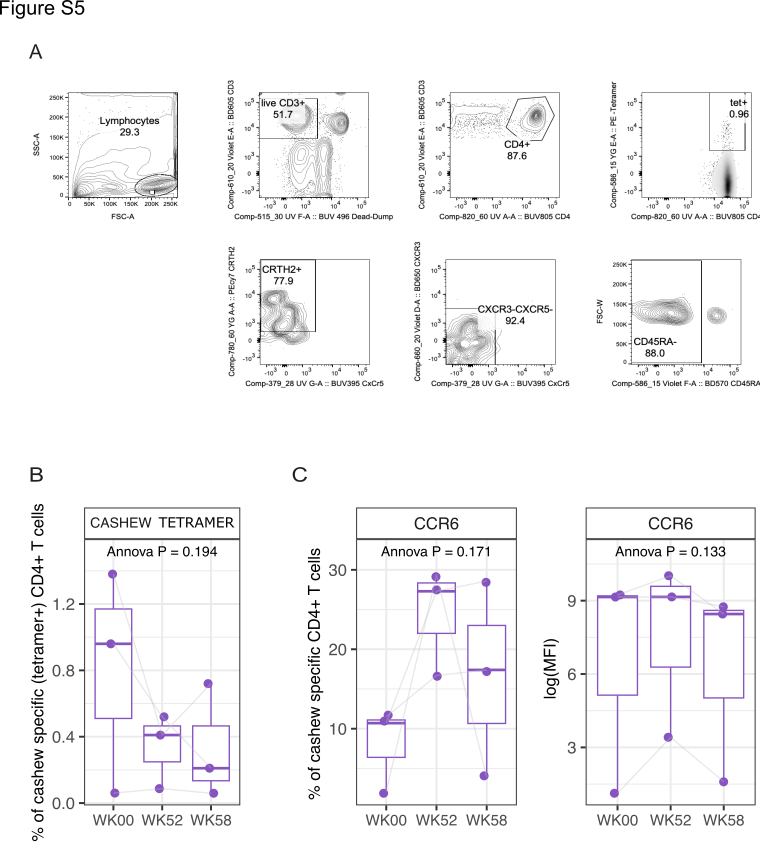
***

***Figure S5:*** (A) Gating scheme for identification of allergen-specific CD4^+^ (Tetramer^+^) T cells from PBMCs. (B) Frequency of allergen-specific CD4^+^ (Tetramer^+^) T cells among total CD4^+^ T cells. (C) Frequency and MFI of CCR6^+^ allergen specific CD4^+^ T cells. For boxplots, p-values were calculated using a repeated measures analysis of variance (ANOVA; adjusted for each sample and batch) to compare differences between groups. The boxplots depict the median, interquartile ranges, and range (whiskers), with outliers omitted to enhance the clarity of the Y-axis range visualization. p-values were adjusted for multiple hypothesis testing using false discovery rate (q-value).

***Figure 6 :*** Cytokine Expression in Cashew Allergy-Plasma


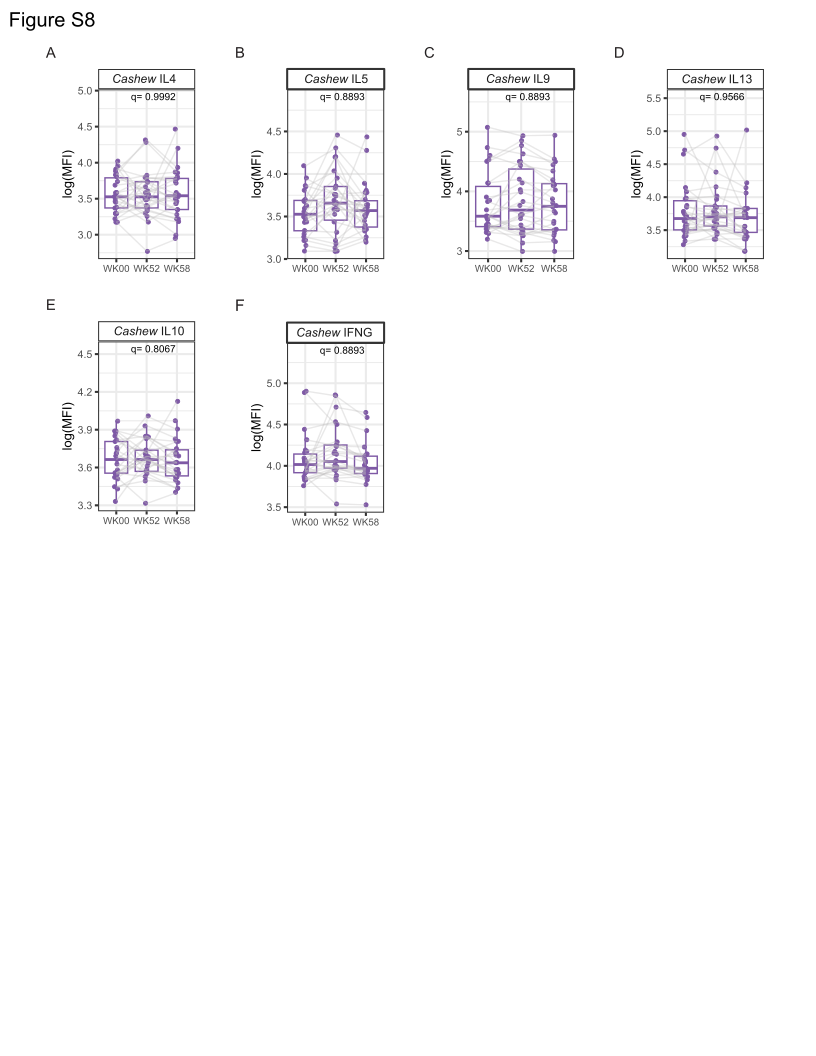


***Figure S6***: Expression of Th2 associated cytokines; (A) IL4 (B) IL5, (C ) IL9 (D) IL13 and Th1 associated (E ) IL10, (F) IFNg in cashew study participants from plasma collected at week 0, week 52, and week 58 that were analyzed using Luminex. For boxplots, p-values were calculated using a repeated measures analysis of variance (ANOVA; adjusted for each sample and batch) to compare differences between groups. The boxplots depict the median, interquartile ranges, and range (whiskers), with outliers omitted to enhance the clarity of the Y-axis range visualization. p-values were adjusted for multiple hypothesis testing using false discovery rate (q-value).

**Figure 7.** Cytokine Expression in Cashew Allergy- Culture Supernatants**
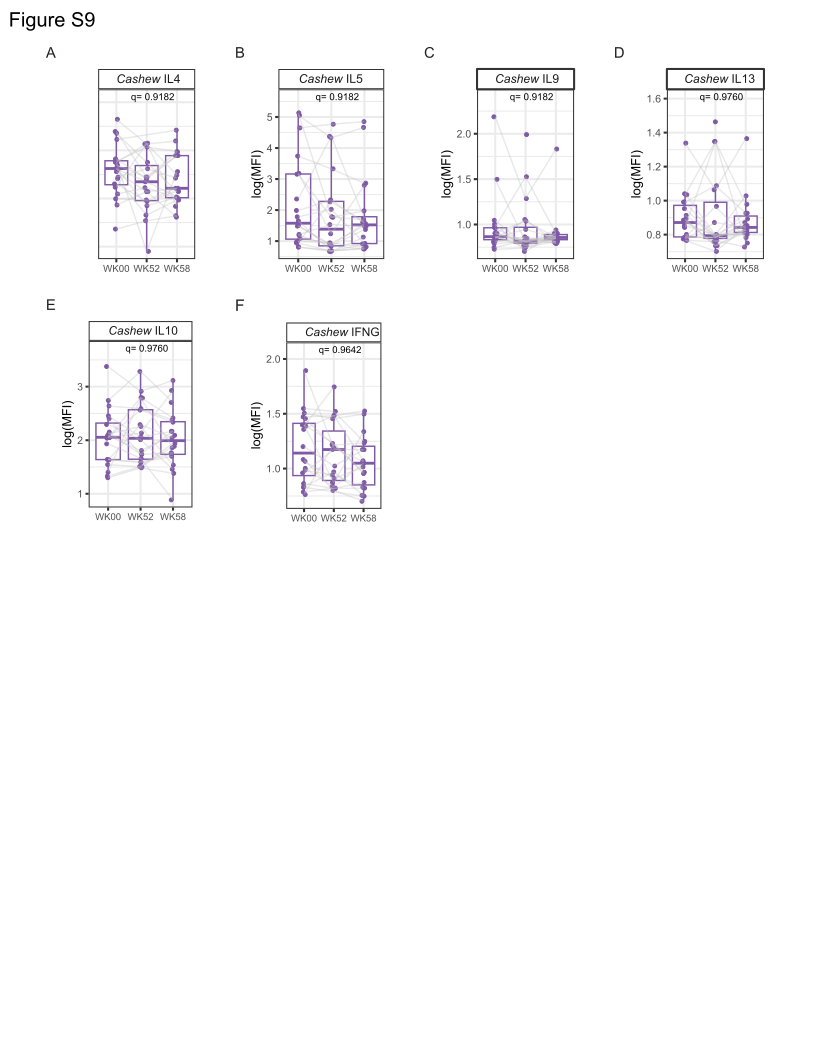
**

***Figure S7***:Expression of Th2 associated cytokines; (A) IL4 (B) IL5, (C ) IL9 (D) IL13 and Th1 associated (E ) IL10, (F) IFNg in cashew study participants from allergen stimulated culture supernatants obtained from PBMCs at week 0, week 52, and week 58 that were analyzed using Luminex. For boxplots, p-values were calculated using a repeated measures analysis of variance (ANOVA; adjusted for each sample and batch) to compare differences between groups. The boxplots depict the median, interquartile ranges, and range (whiskers), with outliers omitted to enhance the clarity of the Y-axis range visualization. p-values were adjusted for multiple hypothesis testing using false discovery rate (q-value).

**Figure S8 Associations between baseline characteristics and AE rate.**


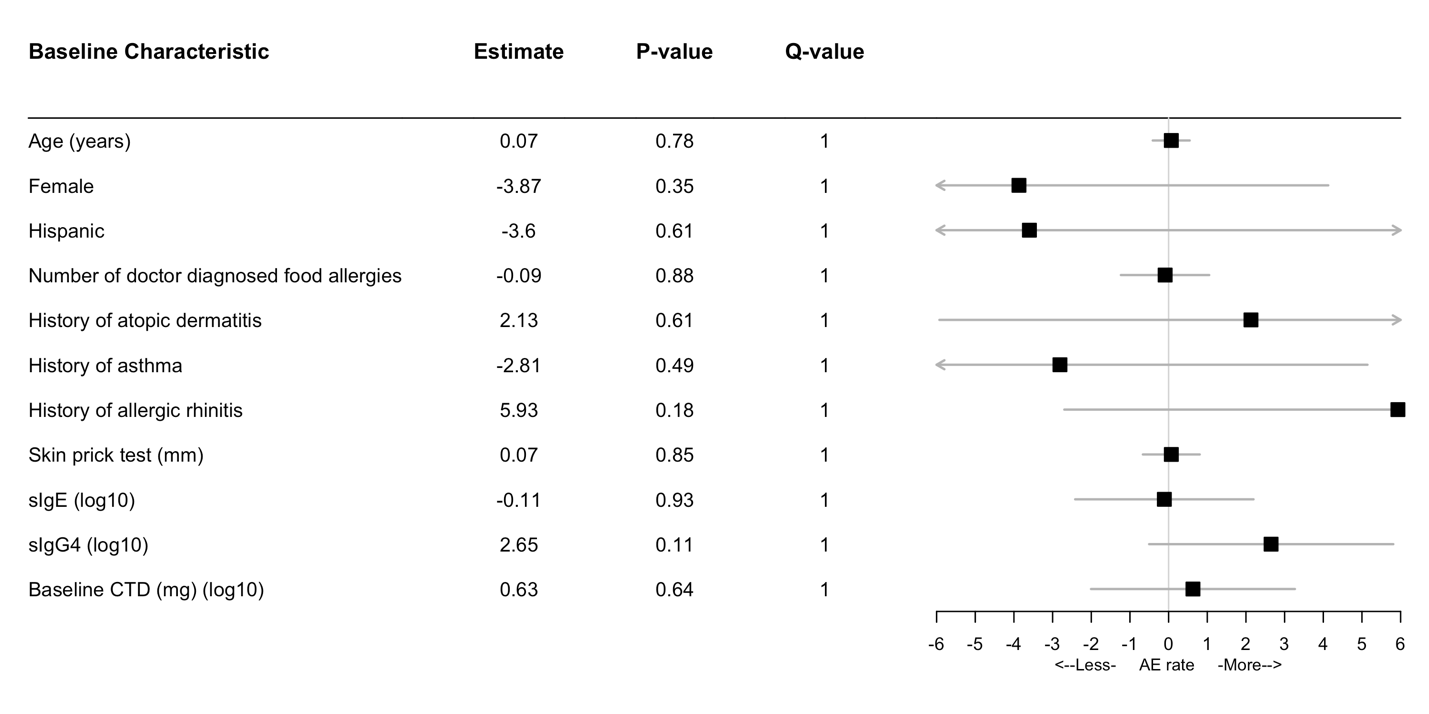


***Figure S8:*** Baseline characteristics and their association with adverse event rate from baseline up to week 52. Each characteristic was fit to a linear regression model adjusting for the allergen group. An estimate above 1 (gray line) denotes an association with a higher AE rate, while an estimate below 1 denotes an association with less. Q-value is the FDR-adjusted P-value.

**Figure S9: Changes in Food Allergy Quality of Life Scores**

1. Changes in Food Allergy Quality of Life Questionnaire – Parent Form

**
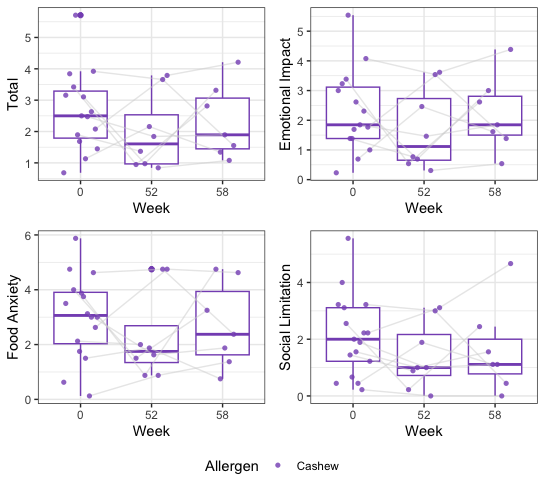
**

1. Changes in Food Allergy Quality of Life Questionnaire (FAQLQ): FAQLQ – Teenage Form (TF), FAQLQ – Adult Form (AF), FAQLQ – Parental Burden (PB)

**
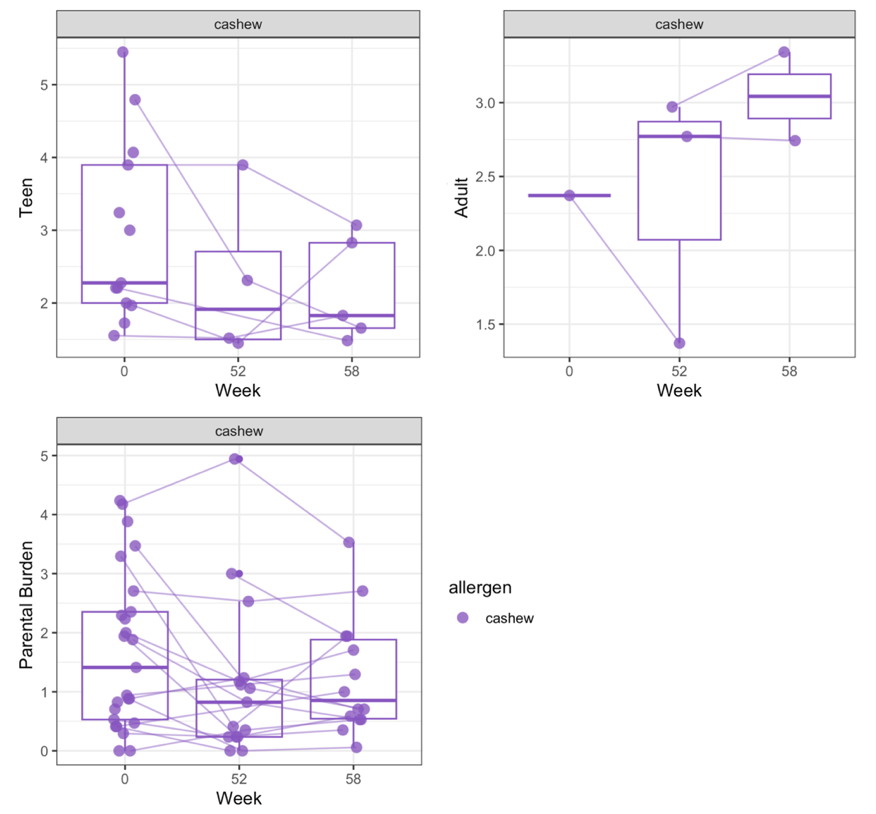
**

***Figure S9:*** Changes in Food Allergy Quality of Life Questionnaire (FAQLQ) (A) FAQLQ-PF by Total Score, Emotional Impact, Food Anxiety and Social Limitation (Week 0 n=18; Week 52 n=8; Week 58 n=8), (B) FAQLQ-TF (Week 0 n=13; Week 52 n=5; Week 58 n=5), FAQLQ-AF (Week 0 n=1; Week 52 n=3; Week 58 n=2), FAQLQ-PB (Week 0 n=27; Week 52 n=15; Week 58 n=15)
